# Modeling Behavior of U-Turning Vehicles at the Median Opening Using a Merging Behavior Approach: A Case Study in Bahir Dar City, Ethiopia

**DOI:** 10.1155/2022/8273616

**Published:** 2022-12-01

**Authors:** Natnael Melsew Wolelaw, Amare Tilahun Tessema, Getachew Asefa Alene

**Affiliations:** ^1^Road and Transport Engineering, Department of Civil Engineering, Debre Tabor University, Debre Tabor, Ethiopia; ^2^Transportation Engineering, Department of Civil Engineering, Debre Tabor University, Debre Tabor, Ethiopia; ^3^Structural Engineering, Department of Civil Engineering, Debre Tabor University, Debre Tabor, Ethiopia

## Abstract

Median openings are one of the most commonly used road features, which are mainly used to allow U-turning movement in urban areas, and this study focuses mainly on modeling the behavior of U-turning vehicles at the median opening using a merging behavior approach. The purpose of the study is to estimate and model the critical gap of u-turning vehicles at the median opening under mixed traffic conditions. Under this study, the accepted gap, rejected gap, driver waiting time, merging time, and critical gap are estimated, and the modified Raff's method and modified INAFOGA method are used for the estimation of a critical gap. However, modified INAFOGA is used for the modeling of critical gaps under mixed traffic conditions. In this study, sixteen median openings were selected in Bahir Dar city, and data were collected using a video recording technique at each selected median opening during the peak hour of the day. The necessary data were extracted using Forevid analysis software tools. Different types of traffic are involved in the mixed traffic, and each vehicle type is categorized according to the Ethiopian Road Authority's 2013 design guide into seven different classes, such as 2-wheeler, 3-wheeler, passenger car, minibus, small bus and truck, medium bus, and medium truck. Among those traffic types, three vehicle classes (three-wheeler, passenger car, and minibus) were only considered due to the prohibition of U-turning movement for medium and large vehicles. For the modeling of critical gaps, waiting time and conflicting traffic flow are used as independent variables using the regression technique. Driver waiting time and the critical gap were found to be power related to passenger cars and minibuses and exponentially to three-wheelers. Conflicting traffic flow and critical gaps were power related to passenger cars and minibuses and linearly related to three-wheelers.

## 1. Introduction

Median openings are one of the most common road facilities used to facilitate the j-turn and U-turn movement of vehicles in Ethiopia, and they are preferred to overpasses and other types of road facilities due to their low cost. Since Bahir Dar is a city located in Ethiopia, median openings are the most common road facilities to facilitate U-turn movement of motorized and nonmotorized vehicles. The majority of signalized intersections do not allow U-turns since doing so would maximize the intersection's conflict spots and enhance its service quality. The most frequently used road facility for U-turn vehicles is a median opening in Bahir Dar due to the city's heterogeneous traffic, which includes a wide variety of vehicle classes. To give U-turn vehicles enough room for safe merging, the through traffic stream is forced to slow down, while U-turn vehicles attempt to enter the median opening. The spaces supplied to U-turning cars are not the normal time headway due to these traffic phenomena, which have an impact on the behavior of the through traffic stream. The entry capacity of the lower priority stream (U-turning vehicles) is significantly impacted by this situation, which also delays the higher priority traffic stream and results in crashes between lower and higher priority traffic streams. This situation forces the acceptance of the gap to be governed by priority rather than natural traffic flow or adherence. Due to all of these factors, median openings are a crash- and delay-prone region. U-turn vehicles at median openings need to be examined by the notion of the parameters which affect the U-turn vehicles at median openings due to the unpredictability of the gap-accepting behavior of U-turn vehicles at median openings.

Therefore, the median opening of separated urban highways is provided to increase intersection efficiency by reducing conflict points, and those openings help provide access to opposing traffic quite well without interfering with priority traffic or U-turn vehicles. To make a safe turn, a U-turning vehicle will always wait for a suitable gap in the subsequent priority traffic stream [1]. Another researcher [2] briefly describes the U-turning behavior of vehicles at the median opening, and the author uses the sectioning method for the study of their behavior and discusses that vehicles enter the U-turn roadway with steadily reduced speed and search the priority traffic stream for a safe exit merging gap at the entry section. On arrival at the exit section, drivers make their intentions known to the priority stream by moving, sometimes menacingly, to the upper end of the exit lane. Priority vehicles may allow the merge to occur, increase speed, change lanes to avoid collusion, flash their headlight, or simply ignore the nonpriority vehicle altogether. At the peak period, merging from the exit point becomes a difficult maneuver and a daring affair.

Due to the high speed, high traffic congestion, and the requirement that the turning vehicle undertake a reverse movement, U-turn movements in median openings are more complicated and dangerous than conventional turning movements at signalized and unsignalized intersections [3]. The turning vehicle must wait and then turn under low-speed conditions in the face of oncoming traffic, which means conflicting traffic may need to accelerate rapidly to achieve the speed of the traffic stream. To analyze the behavior of U-turning movements at the median opening, estimating the critical gap is the most important thing, and as per [4], the critical gap can be defined as the minimum time interval between the through-traffic stream vehicles that is necessary for U-turning vehicles to make a merging maneuver. Gupta et al. [1] state that the values of the critical gap that are accepted vary for different vehicle classes and depend on various parameters, such as the type of U-turning vehicles, several stream parameters of opposing lane traffic, and the geometric elements of the carriageway, including the median.

Different methods have been used by different researchers for a while. For heterogeneous traffic flow conditions, the authors in [5] used existing methods like probit, Hewitt, modified Raff, logit, and Harder methods for the estimation of a critical gap at unsignalized intersections. There was a wide difference (12%–38%) between the critical gap values, which highlighted the limitations of the methods to address mixed traffic situations. Thus, the authors came up with an alternative technique that makes use of the clearing behavior of the driver in conjunction with gap acceptance data. The new method developed in this study was simple and easy to implement under Indian conditions. With this in mind, Pannela and Bhuyan improved the clearing behavior assumed for the unsignalized intersection to the merging behavior of U-turn vehicles at the median opening [6]. This method takes a vehicle's gap-accepting characteristics into account in addition to its actual merging behavior. Assuming merging time shows how a movement is carried out at the median opening. As an alternative, it considers the challenges presented by mixed-traffic conditions, including lane discipline and the rule of priority impact.

This paper aims to estimate and model the critical gap of a U-turning vehicle at the median opening and estimate the critical gap using the merging behavior approach. In addition to estimating the critical gap, we consider the effect of the driver waiting time and volume of conflicting traffic flow on the critical gap of U-turn vehicles.

The paper makes two basic contributions. The first is initiating the study of U-turning vehicles at median openings since the study is new in Ethiopia. This will open the door for other potential researchers to investigate and study more on the U-turning behavior and its impact on traffic streams. The second is estimating and modeling the critical gap of U-turning vehicles under mixed traffic conditions, which will help the transportation agencies fully understand the behavior of the traffic flow at the median opening under U-turn movements.

Related works in different literature studies are discussed in Section 2. The data collection for the estimation of the critical gap, the data extraction as per the merging behavior approach, and the procedure used for the estimation and modeling of the critical gap are discussed in Section 3. In-depth results and discussion obtained from the estimation and modeling of the critical gap are mentioned in Section 4. The conclusions obtained from the detailed results and discussions are mentioned in Section 5, and recommendations based on the findings are discussed in Section 6. Finally, the limitations and gaps of the study are discussed in Section 7.

## 2. Related Works

In this section, we briefly discussed the related work, which focuses on the behavior of U-turning vehicles at the median opening and the estimation and modeling of the critical gap for U-turning vehicles.

### 2.1. Behavior of U-Turning Vehicle Movement

The gap acceptance behavior of U-turn vehicle drivers is highly affected by the waiting time, and the statistical result from the study by Shubber showed that when the driver's waiting time fell in a range between 21 and 30 sec, the driver was forced to accept a gap size less than that, which fell in the range of 11 to 20 sec at a confidence interval of 95%. On the other hand, there is a slight difference in the mean gap acceptance between an interval of (1–10) and (11–20) sec at the same confidence interval [7]. In addition to the drivers' waiting time, another researcher [8] demonstrated that gender has an impact. It was shown that male drivers tend to be more aggressive and take greater risks when doing U-turns. Likewise, commercial drivers were found to be more aggressive than personal vehicles and were found to complete the U-turn using less width of the carriageway in the opposing lane compared to personal vehicles. Furthermore, loaded vehicles are more careful and turn their steering slowly compared to empty vehicles while taking U-turns.

A *U*-turn at a midblock median opening is a complicated maneuver that causes drivers to be unsure whether to accept or reject the available gap in order to prevent a collision with opposing traffic flow. The study by Khan and Mohapatra in 2022 analyzed the dilemma zone for U-turning vehicles at a midblock median opening for different vehicle categories, and they concluded that the dilemma zone would be highly affected by the vehicle size [9].

The study by Khan et al. [10], which focused on estimating the temporal and spatial critical gap of U-turning vehicles at uncontrolled median openings for six different types of U-turning vehicles, revealed that the critical gap values discovered during the research are significantly smaller than those reported in developed countries. This finding illustrates the aggressive driving nature of drivers in developing countries. In 2022, Mazaheri et al. [11] state that the critical gap for drivers of heavy vehicles was nearly lower than that for drivers of cars, indicating that heavy vehicle drivers were acting more aggressively, and another study, which is conducted on the aggressive behavior of drivers at an uncontrolled intersection under mixed traffic conditions, showed that with an increase in the lag/gap value and a decrease in clearing time, the probability of accepting a gap increases. The likelihood also reduces as major road vehicle size grows, whereas minor road vehicle size increases. The lag/gap and speed of major road vehicles decrease, so do the aggression levels of small road vehicles, while they decline as minor road vehicle size and clearing time grow [12].

### 2.2. Development of the Critical Gap Model

The critical gap of a U-turning vehicle at the median opening is highly affected by different parameters. In 2011, El Esawey and Sayed [13] conducted a study on the operational performance of U-turns at the median opening, and they came to the conclusion that the amount of traffic on the major stream has a substantial impact on the outcome of the U-turning maneuver. They also stated that the median openings function well in light to moderate traffic, unless the volume of the competing traffic effect increases to the point where it becomes impossible to make a U-turn.

The merging behavior approach is the most preferable approach to account for the effect of mixed traffic conditions and lane discipline effects on the gap acceptance behavior of drivers, and the critical gap has a strong correlation with the gap between conflicting traffic, the volume of conflicting traffic, and the conflicting traffic speed [4].

As per Datta, critical gaps are strongly correlated with conflicting traffic volume, conflicting traffic speed, and U-turn vehicle waiting times. The model prepared by Datta for four vehicle classes (2W, 3W, 4W, and SUV/MUV) showed that the critical gap has a strong linear correlation with conflicting traffic speed and a strong power correlation with waiting time for each vehicle class and also a strong power relationship with conflicting traffic volume for each vehicle class except for 2W, which has a linear correlation. In 2017, Dash et al. [14] developed a model that correlates the critical gap with the conflicting traffic volume and proved that conflicting traffic has a significant linear relationship with the critical gap of U-turning vehicles.

## 3. Research Methodology

**Figure 1:**
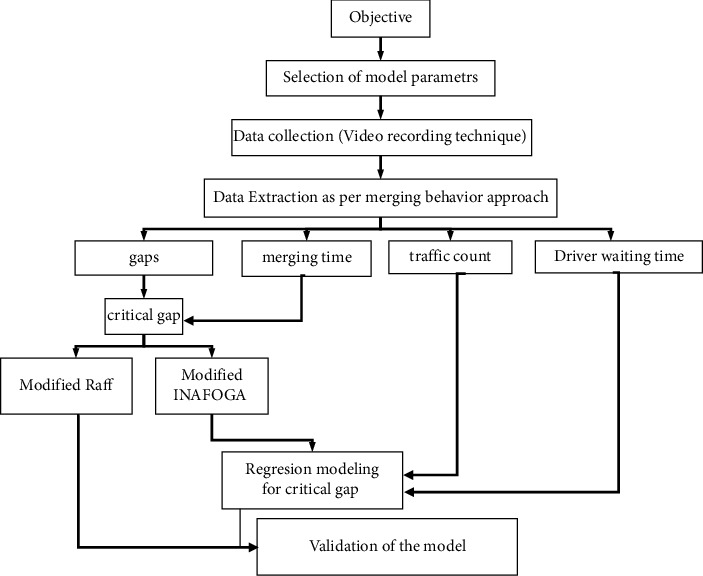
Flowchart of the estimation and modeling of the critical gap.

To analyze the behavior of U-turn vehicles at the median opening, the estimation of the critical gap is the key to a successful design and analysis. However, it is not possible to measure the critical gap directly from the field due to its complexity. Even if it is not possible to measure the critical gap directly from the field, some important parameters can be measured there and help estimate the critical gap. Since accepted gaps and rejected gaps can be measured in the field, we can use them to estimate the critical gaps using different methods. In this study, two approaches, modified Raff's and influence Area for Gap Acceptance (INAFOGA) methods, were used for the estimation of a critical gap, and the procedure used for the estimation and modeling of a critical gap is presented in Figure 1.

### 3.1. Data Collection

**Figure 2:**
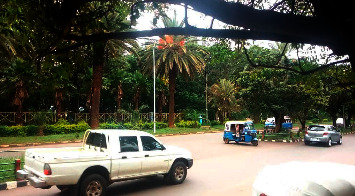
Three-wheel vehicle taking a U-turn at the median opening.

Data collection was performed with the help of video recording techniques at each of the sixteen selected median openings. Recording was performed by fixing the camera on a tripod stand at the top of the building so that we could capture the entire median and its vehicular movement. The data were collected during the peak and off-peak hours of weekdays. Due to the significant variance in data sets, weekend days and public holidays are typically ignored, resulting in inaccurate estimation of important gaps in U-turning traffic near median openings [15]. The data collected through video recording techniques are shown in Figure 2.

### 3.2. Data Extraction as per the Merging Behavior Approach

The video data were analyzed frame by frame with an accuracy of 0.03 s based on a frame rate of 30 frames per second using Forevid analysis software to extract and determine all necessary parameters like merging time, gap acceptance, waiting time, rejected gap, and conflicting traffic volume. Video recording of all sixteen median openings and through vehicles is counted. Through vehicle traffic comprised vehicles including two-wheelers, three-wheelers, passenger cars (pcs), minibuses, small buses, medium buses, small trucks, and medium trucks, and their respective passenger car units are shown in Table 1as classified by the Ethiopian Road Authority [16]. The classes of U-turning vehicles considered in this study are three-wheelers, passenger cars with a seat of fewer than ten passengers, and minibuses with seats between 11 and 14.

Those vehicle classes are considered for this study's purpose as a U-turning vehicle at each selected median opening due to the prohibition of U-turn movement at the median opening by the city municipality; medium and large vehicle classes are not considered as U-turning vehicles.

The merging behavior approach is the most preferable method for the estimation of the critical gap when the traffic condition is mixed, lane discipline is not enforced, and the rule of priority is not obeyed or practiced [3, 6, 17, 18]. The first step of the merging behavior approach is to identify the influence area for gap acceptance (INAFOGA), which is an area, where it is believed that all merging and gap acceptances occur.

#### 3.2.1. Determination of the Influence Area for Gap Acceptance (INAFOGA)

After the video data are recorded and the necessary data related to the geometric future of the median opening are collected, data are extracted to get the different necessary parameters for the estimation and development of the critical gap model. The first thing we need to do for the modeling of the critical gap is identify the INAFOGA.

**Figure 3:**
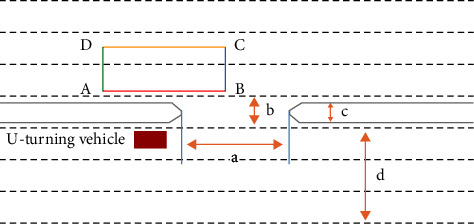
Influence area for gap acceptance (INAFOGA).

As per Mohapatra et al. [19], the identification of the critical position INAFOGA is affected by the size of the vehicle, and it is shown that a smaller vehicle has a large standard deviation compared to larger vehicles on the uniqueness of the INAFOGA. In the identification of INAFOGA, different researchers have recommended different methodologies, but as per [3, 19], the authors use the merging behavior approach for the identification. In this approach, the cumulative clearing time was calculated as the time required by a lower-priority vehicle to clear the INAFOGA, the curve was plotted, and the cumulative frequency of the corresponding lag and gap acceptance was calculated for each vehicle category after the intersection of the curves, which is considered the critical gap. The identification of INAFOGA is performed, as shown in Figure 3.

After a careful look at the video data collected at each specific median opening, the position of each line of the INAFOGA is determined as follows:

- Upper Boundary (Line AB): it is a reference line for the assessment of service delay, and this line is the stopping line of U-turning vehicles before they merge with conflicting traffic or wait for the right acceptable gap. This line is considered the reference point for the assessment of merging movements.
- Downstream Boundary (Line DA): it is a position in which the U-turning vehicle completely merges with conflicting traffic, and its position will vary according to the size of the U-turning vehicle and is sometimes called the merging line.
- Upstream Boundary (Line BC): it is considered to be at a distance of half the median width, and this line will be used as the reference line for the extraction of the time headways of conflicting traffic.
- Lower Boundary (Line CD): this line is identified by studying the critical position of the U-turning vehicle.

However, the authors in [3] state that the identification of upstream boundary BC and lower boundary CD will have less significance in estimating the merging time of the U-turning vehicle.

After the influence area for gap acceptance (INAFOGA) is identified, the basic data for the estimation of the critical gap using the modified INAFOGA and modified Raff's methods are extracted.

The gap is estimated as the time difference between the arrival of the consecutive through vehicle and line BC. The merging time is the time taken to traverse the INAFOGA without causing any crashes with conflicting traffic, and it is measured from the time the rear bumper passed over stop line AB to the time the rear bumper crossed merging line AD. Driver waiting time is the time the U-turning vehicle spends waiting for the gap sufficient for full merging after he/she reaches the stop line of the INAFOGA.

### 3.3. Estimation of Critical Gaps

The critical gap (*t*_*c*_) is the minimum time interval between the through-traffic stream vehicles that is necessary for a U-turning vehicle to make a merging maneuver. The value of the critical gap can differ, depending on the driver's behavior, which is why some drivers accept a smaller gap and others accept a too long gap based on the driver's behavior.

Different methods have been used throughout the year for the estimation of the critical gap since it cannot be measured directly in the field. However, there are many different methods used for the estimation of critical gaps. Among those methods, the modified INAFOGA method is the one that is designed to address the mixed traffic condition under lane discipline and the rule of priority effects, and due to this, the method is used for the modeling of the critical gap. The modified Raff's method is used for the comparison of the model results in the validation process.

#### 3.3.1. Modified Raff's Method

The earliest method for estimating the critical gap was first proposed by Brilon et al. [20], and the authors defined that the critical gap *t*_*c*_ is the value of time *t* which is a function of

(1) $$\begin{array}{c} 1-F_{r}\left(t\right)andF_{a}. \end{array}$$

This method involves the empirical distribution functions of accepted gap *F*_*a*_ (*t*) and rejected gap *F*_*r*_ (*t*). As per the Raff method, a critical gap at unsignalized intersections is defined as “the gap/lag for which no. of accepted gaps shorter than it is equal to the no. of rejected gaps longer than it.” The arrival of mainstream vehicles can be described by a Poisson distribution but only for light-medium traffic flow conditions. Raff's method involves the extraction of the length of the gaps in seconds, for which the driver waits at the median opening to accept a suitable gap, an accepted gap, or a rejected gap. Two cumulative distribution curves will be drawn with no gaps in the ordinate and gap size as abscissa, and these two cumulative distribution curves will relate gap length (*t*) with the accepted gap and rejected gap. After the curves are drawn, the intersection of the two cumulative curves will be taken as a critical gap for that specific vehicle type [21–23].

#### 3.3.2. Modified INAFOGA Method

The new method developed in this study was simple and easy to implement under Indian conditions. With due consideration, this paper has provided significant background for the present study because of its heftiness towards mixed traffic conditions prevailing in Ethiopia. The “clearing behavior” assumed for unsignalized intersections in the previous study was improved to the merging behavior in the case of U-turn vehicles at median openings in this study, as [14, 17, 24, and 25] used in their study. It considers the actual merging behavior in addition to the gap acceptance features of a vehicle. Merging time shows how the movement is implemented at the median opening. It also takes into account the difficulties found under mixed traffic conditions.

In this method, a cumulative distribution graph that contains the gap size on the *x*-axis and the cumulative percentage of gaps on the *y*-axis and two cumulative percentage graphs of the merging time and accepted gap will be prepared, and the intersection of the two will be taken as the critical gap of the specific vehicle class.

### 3.4. Modeling of the Critical Gap

Modeling of the critical gap is performed as per the merging behavior approach, and the critical gap estimated using the modified INAFOGA method was used as the dependent variable. Independent variables are first selected based on the effect they have on the estimated critical gap value as per different research, and researchers have identified conflicting traffic flow, conflicting traffic speed, width and number of the lanes, the geometric feature of the median opening, and drivers' waiting time as having a major impact on the estimation of the critical gap value and mainly affecting the driver's decision. Among those parameters, conflicting traffic flow and waiting time were considered the major parameters for the modeling of the critical gap. Since the critical gap is estimated based on the merging behavior approach, the merging time and accepted gap are the variables used for the estimation of the critical gap.

## 4. Results and Discussion

### 4.1. Critical Gap Estimation

**Figure 4:**
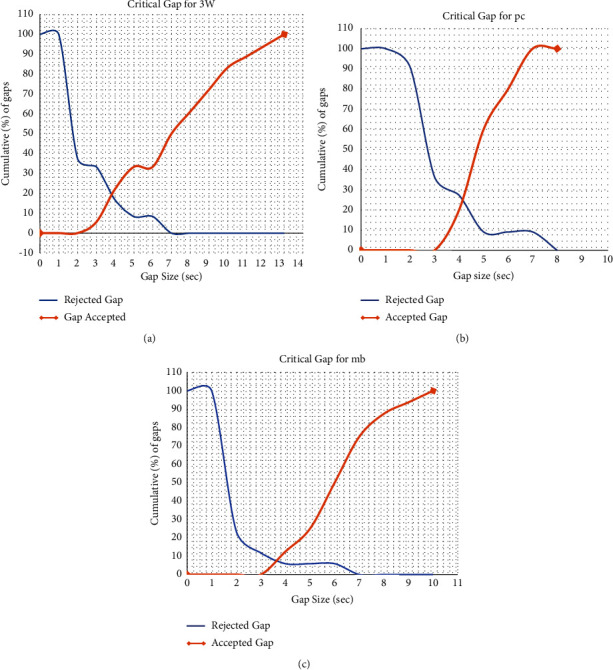
Modified Raff's method estimation result. (a) Three-wheeler, (b) passenger car, and (c) minibus.

**Figure 5:**
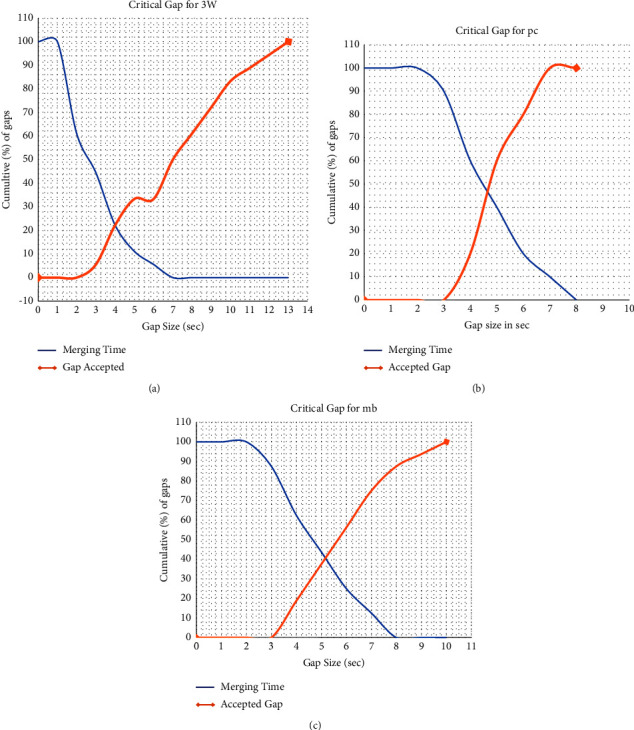
Modified Raff's method estimation results for 3W, pc, and mb, respectively. (a) Three-wheeler, (b) passenger car, and (c) minibus.

- Modified Raff's method: the critical gap for all data points in each median is estimated as the number of accepted lags shorter than the critical lag and is the same as the number of accepted lags longer than the critical lag [23]. The critical gap for each U-turning vehicle (3W, pc, and mb) is estimated, as shown in Figure 4.
- Modified INAFOGA method: after the merging time and accepted gap data are extracted from the video recorded according to the merging behavior approach, the cumulative frequency distribution curve of the merging time and accepted gap will be plotted, and the point of the intersection of the two curves at the *x*-axis will be taken as the critical gap of the specific vehicle class. The result of the merging modified INAFOGA is shown in Figure 5.

The summarized result of the critical gap estimation using both methods is presented in Table 2 for each of the three U-turning vehicle classes at each of the sixteen median openings.

### 4.2. Modeling of the Critical Gap

The critical gap of U-turning vehicles at the median opening is modeled in terms of the driver waiting time and conflicting traffic flow. Driver waiting time is the time that the U-turning vehicle spent waiting for the gap sufficient for full merging after he/she reached the stop line of the INAFOGA, and it will be extracted from the video recording data from each selected median opening. The conflicting traffic flow is counted from the video recorded, and the hourly traffic volume is converted using their respective passenger car units (pcus) as recommended by the Ethiopian Road Authority [16]. Seven different types of vehicle classes were involved in our study, as shown in Table 1.

The correlation for the development of the regression model was performed between the critical gap and waiting time and the critical gap and conflicting traffic flow for the U-turning vehicle class.

#### 4.2.1. Critical Gap vs Driver Waiting Time Model

It has been shown in different research studies that waiting time varies from driver to driver based on their age, sex, and other behaviors according to the gap they accept [26]. Different studies proved that the waiting time affects the critical gap at unsignalized intersections as well as at median openings, and as the waiting time increases, the critical gap becomes smaller, which is due to the driver's loss of patience for waiting and accepting the smaller gap than the one they rejected before. The waiting time for the modeling of the critical gap is taken as the average waiting time of every hour for modeling.

**Figure 6:**
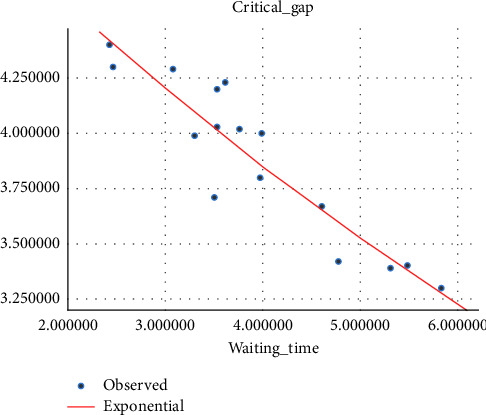
Variation of the critical gap with the driver's waiting time for three-wheelers.

**Figure 7:**
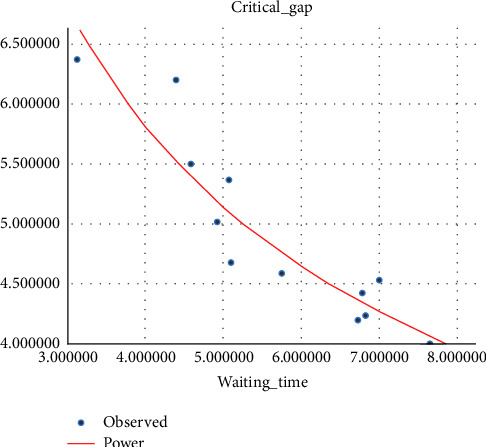
Variation of the critical gap with the driver's waiting time for passenger cars.

**Figure 8:**
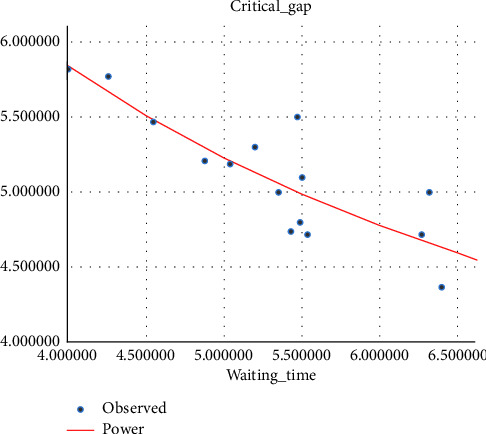
Variation of the critical gap with the driver's waiting time for mini-bus.

(i) Three-wheeler (3W) vehicle: in the regression model, the exponential curve fitting shows a good *R*-square fit to the relationship between the critical gap and drivers' waiting time, and in this relationship, as the drivers' waiting time increases, drivers lose patience and tend to accept smaller gaps. The variation of the critical gap with driver waiting time is shown in Figure 6.
  (2) $$\begin{array}{c} t_{c}=5.473e^{-0.088t_{w}}\ldots with\;R^{2}=0.86\text{.} \end{array}$$
(ii) Passenger car (pc): the curve-fitting result has shown that for the vehicle category passenger car, the correlation between the critical gap and the driver waiting time is a power relationship, as shown in Figure 7.
  (3) $$\begin{array}{c} t_{c}=12.508{t_{w}}^{-0.553}\;\ldots with\;R^{2}=0.868\text{.} \end{array}$$
(iii) Minibus (mb): the model result showed that the critical gap and driver waiting time is power related to a good fit *R*-square, as shown in Figure 8.
  (4) $$\begin{array}{c} t_{c}=11.6{t_{w}}^{-0.495}\ldots with\;R^{2}=0.7\text{.} \end{array}$$

#### 4.2.2. Critical Gap vs Conflicting Traffic Flow

Sometimes the U-turning maneuver may be taken in a jam condition without any sufficient gap [27, 28], and this sometimes leads to collisions. Different research studies have proved that conflicting traffic flow has a significant effect on the estimation of the critical gap, and due to this, different models have been prepared by so many researchers in the past that show the correlation between conflicting traffic flow and the critical gap [19, 29, 30].

**Figure 9:**
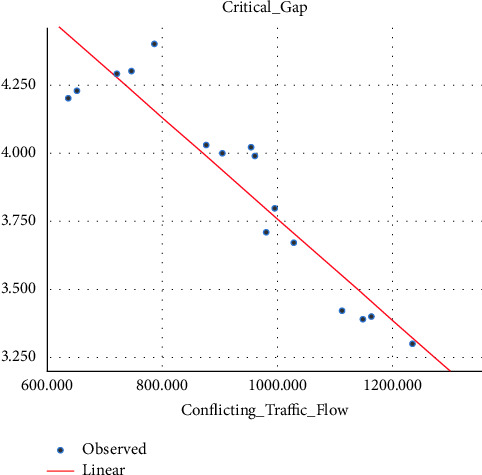
Variation of the critical gap with conflicting traffic flow for three-wheelers.

**Figure 10:**
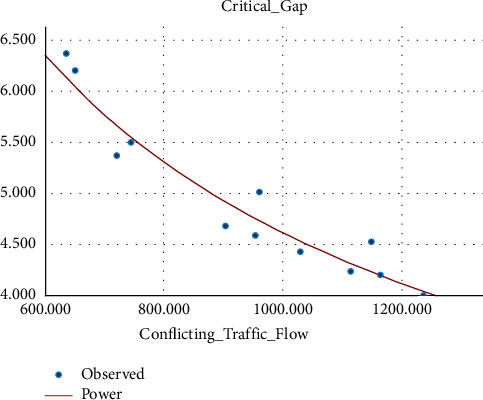
Variation of the critical gap with conflicting traffic flow for passenger cars.

**Figure 11:**
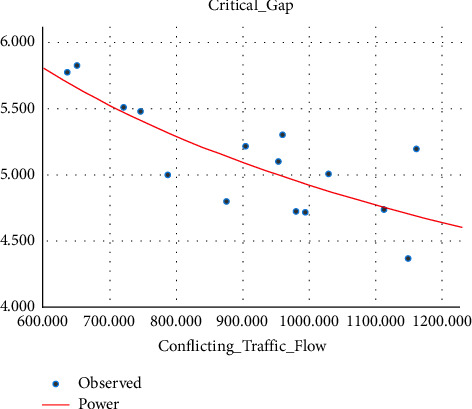
Variation of the critical gap with conflicting traffic flow for three-wheelers.

(i) Three-wheeler (3W): the critical gap of a U-turning 3W vehicle is correlated with the average hourly conflicting traffic flow and their relation fitted into some mathematical models. The model showed that the critical gap and conflicting traffic flow are linearly related, as indicated by the best fit *R*-square. The variation of the critical gap with conflicting traffic flow is shown in Figure 9.
  (5) $$\begin{array}{c} t_{c}=-0.002Q_{t}+5.608\ldots with\;R^{2}=0.875\text{.} \end{array}$$
(ii) Passenger car (pc): unlike three-wheeler vehicle classes, the relation between the conflicting traffic flow and the critical gap has a power relationship, and as the conflicting traffic flow increases, the critical gap decreases due to the small size gap provided for U-turning vehicles. The relationship between the critical gap and conflicting traffic flow is shown in Figure 10.
  (6) $$\begin{array}{c} t_{c}=357.664{Q_{t}}^{-0.63}\ldots with\;R^{2}=0.93\text{.} \end{array}$$
(iii) Minibus (mb): the correlation between the conflicting traffic flow and critical gap for a minibus is best fitted through the power relationship of the model, and as the conflicting traffic flow increases, the critical gap value reduces. The model is best fitted with an *R*-square value of 0.61, as shown in Figure 11.
  (7) $$\begin{array}{c} t_{c}={{45.526Q}_{t}}^{-0.322}\ldots with\;R^{2}=0.61\text{.} \end{array}$$

### 4.3. Validation of the Model

For the validation of the model developed, new four median openings are selected, and the critical gap is calculated using the modified Raff's method and the model developed. The result from the model is compared with the modified Raff's method result to check if there is a significant difference between them using an independent sample *t*-test (Levene's test for equality of variance and *t*-test for equality of means). The result from the independent sample *t*-test at a 95% confidence interval showed there is no significant difference between the model value and the modified Raff's method value, in which the *p* value from Levene's test and the *t*-test is greater than the significance level (0.05). As there is no significant difference between them as shown in Tables 3–8, the null hypothesis will be accepted.

## 5. Conclusion

The present study estimates the critical gap using both modified Raff's and modified INAFOGA methods, and the merging behavior approach is used as the base approach for the modeling of the critical gap since the approach can account for the impact of mixed traffic conditions, violations of rules of priority, and lane discipline. The modified Raff's method result showed that the maximum and minimum critical gaps for three-wheelers are 4.4 and 3.3 seconds, 6.38 and 4 seconds for passenger cars, and 5.82 and 4.37 for minibuses, respectively. The result showed that three-wheelers take smaller available critical gaps due to their smaller size, while passenger cars take the higher critical gap due to a lack of driving experience in comparison with minibuses. Since most passenger cars are used for private use, minibuses are used as a taxi mode of the transport system, andas a result of this, passenger car drivers tend to accept larger gap due to their lack of driving experience. In the modified INAFOGA method, the maximum and minimum critical gaps for three-wheelers are 4.77 and 2.77 seconds, for passenger cars, they are 5.51 and 3.43 seconds, and for minibuses, they are 7.01 and 3.49 seconds, respectively. For critical gap modeling, two independent variables (drivers' waiting time and the conflicting traffic flow) were taken into account. The critical gap and those independent variables, whose probability value is less than the significance level (5%), were empirically related, using the regression technique for each U-turning vehicle, and the model result showed a high coefficient of determination (*R*^2^). From the model result, conflicting traffic flow has an inverse relationship with the critical gap, and as the volume of conflicting traffic flow increases, U-turning drivers accept smaller gaps. The drivers' waiting time has an exponential correlation with the critical gap and a linear correlation with the conflicting traffic flow for three-wheeler vehicles. For passenger cars and minibuses, both drivers' waiting time and conflicting traffic flow showed a power relationship with the critical gap.

It is important to note that there are many factors to be considered in the study of the U-turn behavior of the vehicle, such as the geometric feature of the median opening, the gender of the driver, and the traffic management system. Further research needs to be performed on the effect of those factors on the critical gap of U-turning vehicles at the median opening.

## 6. Recommendations

The result of this study may be used for the analysis and modeling of the capacity of median openings so that U-turning traffic movement becomes safe and efficient. In addition to this, the study can be used in the study of the effect of U-turning vehicles on the flow of the major traffic stream and in delay and queue analysis.

## 7. Limitations

This study looks into the gap acceptance behavior of U-turning vehicles at the median opening and the effect of conflicting traffic flow and drivers' waiting time on the critical gap. However, even if the critical gap is highly affected by these parameters, it is also affected by the geometric feature of the road width and conflicting traffic speed. It is certain that the speed of conflicting traffic speed and geometric features of the road highly influence the critical gap of U-turning vehicles, which is proposed to be investigated in a future study. The effect of driver experience, gender, and age is also proposed to be studied in feature work.

## Figures and Tables

**Table 1:** Passenger car units for each vehicle class.

Vehicle class	PCU factor
2-Wheeler (motorized)	0.63
3-Wheeler	0.82
Passenger car	1
Minibus	1.19
Small bus and truck	1.4
Medium bus	1.67
Medium truck	1.67

**Table 2:** Summary of the critical gap estimated.

MO	Modified INAFOGA			Modified Raff's		
	3W	pc	mb	3W	pc	mb
1	3.85	4.18	3.49	4	4.68	5.21
2	3.82	3.83	4.9	4.02	4.59	5.1
3	4.32	—	5.12	3.8	—	4.72
4	4.47	5.51	7.01	3.42	4.24	4.74
5	3.87	4.62	4.43	4.23	6.21	5.82
6	3.25	4.35	4.68	4.2	6.38	5.77
7	3.68	4.22	5.16	4.3	5.51	5.47
8	3.55	4.05	4.35	4.29	5.37	5.5
9	4.17	3.43	4.19	3.4	4.2	5.19
10	3.47	—	5.06	4.4	—	5
11	4.32	—	4.88	3.71	—	4.72
12	3.71	—	4.28	4.03	—	4.8
13	2.9	3.44	—	3.3	4	—
14	3.07	3.94	3.7	3.67	4.43	5
15	4.72	4.2	4.36	3.39	4.53	4.37
16	4.77	5.21	5	3.99	5.02	5.3

**Table 3:** Independent sample *t*-test result of the critical gap vs driver waiting time of the model for three-wheelers.

Independent sample *t*-test		Critical gap	
		Equal variances assumed	Equal variances not assumed
Levene's test for equality of variances	*F*	0.661	
	Sig.	0.447	
*t*-test for equality of means	*t*	0.247	0.247
	d*f*	6	4.861
	Sig. (2-tailed)	0.813	0.815

**Table 4:** Independent sample *t*-test result of the critical gap vs conflicting traffic flow of the model for three-wheelers.

Independent sample *t*-test		Critical gap	
		Equal variances assumed	Equal variances not assumed
Levene's test for equality of variances	*F*	0.029	
	Sig.	0.817	
*t*-test for equality of means	*t*	−1.844	−1.844
	d*f*	6	5.776
	Sig. (2-tailed)	0.115	0.117

**Table 5:** Independent sample *t*-test result of the critical gap vs driver waiting time of the model for passenger cars.

Independent sample *t*-test		Critical gap	
		Equal variances assumed	Equal variances not assumed
Levene's test for equality of variances	*F*	0.265	
	Sig.	0.625	
*t*-test for equality of means	*t*	-2.334	-2.334
	d*f*	6	5.412
	Sig. (2-tailed)	0.056	0.058

**Table 6:** Independent sample *t*-test result of the critical gap vs conflicting traffic flow of the model for passenger cars.

Independent sample *t*-test		Critical gap	
		Equal variances assumed	Equal variances not assumed
Levene's test for equality of variances	*F*	0.381	
	Sig.	0.56	
*t*-test for equality of means	*t*	−3.34	−3.34
	d*f*	6	5.995
	Sig. (2-tailed)	0.052	0.054

**Table 7:** Independent sample *t*-test result of the critical gap vs driver waiting time of the model for minibuses.

Independent sample *t*-test		Critical gap	
		Equal variances assumed	Equal variances not assumed
Levene's test for equality of variances	*F*	4.43	
	Sig.	0.08	
*t*-test for equality of means	*t*	−1.715	−1.715
	d*f*	6	5.221
	Sig. (2-tailed)	0.137	0.145

**Table 8:** Independent sample *t*-test result of the critical gap vs conflicting traffic flow of the model for minibuses.

Independent sample *t*-test		Critical gap	
		Equal variances assumed	Equal variances not assumed
Levene's test for equality of variances	*F*	0.318	
	Sig.	0.56	
*t*-test for equality of means	*t*	−3.34	−3.34
	d*f*	6	5.995
	Sig. (2-tailed)	0.051	0.053

## Data Availability

The data that support the findings of this study are available from the corresponding author upon request.

## Abbreviations

MO: Median opening
*t*: The time headway between successive vehicles in the major stream (second)
*t *_*c*_: Critical gap (second)
INAFOGA: Influence Area for Gap Acceptance
2W: Two-wheeler
3W: Three-wheeler
4W: Four-wheeler
pc: Passenger car
SUV: Sport utility vehicle
MUV: Multiutility vehicle
mb: Minibus.
